# SENP1-mediated deSUMOylation of JAK2 regulates its kinase activity and platinum drug resistance

**DOI:** 10.1038/s41419-021-03635-6

**Published:** 2021-04-01

**Authors:** Jing Li, Ruiqin Wu, Mingo M. H. Yung, Jing Sun, Zhuqing Li, Hai Yang, Yi Zhang, Stephanie S. Liu, Annie N. Y. Cheung, Hextan Y. S. Ngan, John C. Braisted, Wei Zheng, Huiqiang Wei, Yingtang Gao, Peter Nemes, Huadong Pei, David W. Chan, Yiliang Li, Wenge Zhu

**Affiliations:** 1 Department of Biochemistry and Molecular Medicine, The George Washington University School of Medicine and Health Sciences, Washington, DC 20037 USA; 2 GW Cancer Center, The George Washington University, Washington, DC 20052 USA; 3 Department of Obstetrics and Gynecology, LKS Faculty of Medicine, The University of Hong Kong, Hong Kong SAR, China; 4 National Center for Advancing Translational Sciences, National Institutes of Health, Bethesda, MD 20892 USA; 5 Tianjin Key Laboratory of Radiation Medicine and Molecular Nuclear Medicine, Institute of Radiation Medicine, Peking Union Medical College & Chinese Academy of Medical Sciences, Tianjin, 300192 China; 6 Key Laboratory of Artificial Cell, Institute for Hepatobiliary Disease, Tianjin Third Central Hospital, Tianjin, 300170 China; 7 Department of Chemistry and Biochemistry, University of Maryland, College Park, MD 20742 USA

**Keywords:** Proteases, Cancer therapeutic resistance

## Abstract

The JAK2/STAT pathway is hyperactivated in many cancers, and such hyperactivation is associated with a poor clinical prognosis and drug resistance. The mechanism regulating JAK2 activity is complex. Although translocation of JAK2 between nucleus and cytoplasm is an important regulatory mechanism, how JAK2 translocation is regulated and what is the physiological function of this translocation remain largely unknown. Here, we found that protease SENP1 directly interacts with and deSUMOylates JAK2, and the deSUMOylation of JAK2 leads to its accumulation at cytoplasm, where JAK2 is activated. Significantly, this novel SENP1/JAK2 axis is activated in platinum-resistant ovarian cancer in a manner dependent on a transcription factor RUNX2 and activated RUNX2/SENP1/JAK2 is critical for platinum-resistance in ovarian cancer. To explore the application of anti-SENP1/JAK2 for treatment of platinum-resistant ovarian cancer, we found SENP1 deficiency or treatment by SENP1 inhibitor Momordin Ic significantly overcomes platinum-resistance of ovarian cancer. Thus, this study not only identifies a novel mechanism regulating JAK2 activity, but also provides with a potential approach to treat platinum-resistant ovarian cancer by targeting SENP1/JAK2 pathway.

## Introduction

Janus kinase 2 (JAK2) is one of the JAK family of intracellular non-receptor tyrosine kinases consisting of 4 mammalian members, JAK1, JAK2, JAK3, and TYK2^1^. JAK2 acts as a critical cytoplasmic signaling component of cytokine receptors, as such participates in a variety of cellular responses important for inflammation, growth, metabolism, drug resistance as well as gene transcription^1–4^. JAK2 has emerged as a promising target in myeloproliferative neoplasms, and increasingly in solid tumors, such as lung cancer, breast cancer and gastric cancer^5–10^. A single mutation V617F of JAK2 is seen in more than 90% of patients with polycythemia vera and in nearly 50% of patients with primary myelofibrosis and essential thrombocythemia^11–13^. This mutation leads to constitutive activation of JAK2^13^, which is sufficient to drive myeloproliferative disorders in mouse models.

JAK2 is recruited and activated by ligand binding with cytokine-activated receptors on cellular membrane^4,14^. The canonical action of JAK2 is to activate the signal transducers and activators of transcription (STAT) proteins in cytoplasm, which then translocate to the nucleus to initiate specific transcriptional programs^15,16^. Our recent study indicated that JAK2/STAT/anti-apoptosis signaling is responsible for platinum-resistance in ovarian cancer^4^. The role of JAK2 has been restricted in cytoplasm as the activator of the STATs for decades. However, over the last several years, numerous studies have demonstrated the presence of JAK2 inside the nucleus to regulate the transcription factors other than STATs, or to act as the epigenetic regulators^17^. Therefore translocation of JAK2 between nucleus and cytoplasm is an important mechanism to regulate cellular function of JAK2^18^. However, how JAK2 translocation is regulated and what is the physiological function of JAK2 cellular translocation remain largely unknown.

Small ubiquitin-related modifier (SUMO) is an ubiquitin-like protein moiety reversibly added post-translationally to target proteins by an ATP-driven cascade of enzymes consisting of activating enzyme (E1), conjugating enzyme (E2), and ligase (E3)^19,20^. SUMOylation has been found to regulate many cellular events including chromatin organization, transcription, DNA damage repair, protein trafficking, and signal transduction^21,22^. Like ubiquitin, SUMO can be attached as a single entity to the ε-amino group of a lysine residue on the substrate protein or SUMO itself to form a multiunit chain. SUMO moieties on modified proteins can be removed by SUMO-specific proteases called sentrin-specific proteases (SENPs)^23,24^, which include SENP1, SENP2, SENP3, SENP5, SENP6 and SENP7^25^. SENP1 is a nuclear protease that has been shown to deSUMOylate several targets, including hypoxia-inducible factor-1α (HIF1α), histone deacetylase 1 and androgen receptor^26–28^. SENP1 expression level is increased in human testis, breast, cervical cancer and others^29^. Therefore, targeting SENP1 has been regarded as a useful approach for cancer therapy. During last several years, a few SENP1 specific inhibitors have been developed, but potent inhibitors have yet to be identified^30–35^.

In this study, for the first time, we discovered that SENP1 directly regulates JAK2 cellular localization via deSUMOylation, and activated SENP1/JAK2 signaling is critical for platinum-resistance in ovarian cancer. Our clinical studies demonstrated that SENP1/JAK2 signaling is activated in tumors from platinum-resistant ovarian cancer patients. To explore the clinical application of anti-SENP1 for the treatment of platinum-resistant ovarian cancer patients, we found that SENP1 siRNA or inhibitor Momordin Ic exhibits remarkable synergistic effect with cisplatin on platinum-resistant ovarian cancer. Thus, inhibition of SENP1 is a promising strategy for the treatment of platinum-resistant ovarian cancer.

## Methods and materials

### Antibodies and reagents

Antibodies used for Western blot, IHC, and IF are: Anti-SENP1 (ab108981; Abcam), JAK2 (3230 S; Cell Signaling Technology), SUMO2/3 (ab3742; Abcam), STAT3 (ab5073; Abcam), p-STAT3 (ab76315; Abcam), Bcl-xL (2764 S; Cell Signaling Technology), and β-actin (5441; Sigma-Aldrich).

Cisplatin (1134357; Sigma) was dissolved in sterile saline for cell survival and xenograft studies. Momordin Ic (A14773; Adooq Bioscience) was dissolved in DMSO.

### Cell culture and resistance cell line establishment

IGROV1 cells (a generous gift form Wei Zheng), U2OS (HTB-96; ATCC), HEK293T (CRL-11268; ATCC) and SKOV3 cells (HTB-77; ATCC) were cultured at 37 °C in DMEM with 10% Fetal Bovine Serum (FBS). PEO1 (10032308; Sigma-Aldrich), PEO4 (10032309; Sigma-Aldrich), PEO14 (10032311; Sigma-Aldrich) and PEO23 cells (10032313; Sigma-Aldrich) were cultured at 37 °C in RPMI-1640 with 10% FBS. OV90 (CRL-11732; ATCC) was cultured in medium containing 1:1 MCDB 105 (Sigma) and M199 (Sigma) supplemented with 10% FBS at 37 °C. All the cells were cultured at in a humidified incubator with 5% CO_2_ atmosphere.

Cisplatin resistant cell lines, including SKOV3 CR, IGROV1 CR, and OV90 CR, were generated using approach as we previously reported^4^.

### Mass spectrometry

Proteins were stained in SDS-PAGE gels with Coomassie blue. Gel lanes were subsequently sliced and digested in-gel overnight at 37 °C with trypsin. Peptides were eluted in 40% of acetonitrile with re-suspension in 20 μl of 2% formic acid before second extraction. Samples were then dried in a Savant SpeedVac, and resuspended in a 5% methanol/0.1% formic acid solution. Tryptic peptides were separated on C18 reverse phase columns, and were analyzed by Thermo Proteome Discoverer (version 2.1.1.21, Thermo). Proteins were identified by using the Mascot (version:2.3.01) search engine against the Homo sapiens (Human)-Uniprot (TrEMBL) protein databases.

### In vitro SUMOylation assay

The SUMOylation reactions were performed according to the manufacture’s instruction (BML-UW8955-0001; ENZO). Briefly, 200 nM of purified HIS-JAK2 was suspended in reaction buffer (20 μl) containing Mg-ATP, SUMO E1, SUMO E2 and recombinant SUMOs. RanGAP1 (provided in the kit) was used as the substrate for positive control. The reaction was incubated at 37 °C for 60 min and terminated by using 20 μl of SDS loading buffer. For deSUMOylation assay, SENP1 was added 60 min after SUMOylation reaction and incubated for additional 60 min. The reactions were then subjected to Western blot to evaluate SUMOylation level.

### Protein expression and purification

pET28a-JAK2 and its mutant was transformed in BL21 (DE3) *E. coli* cells by heat shot method at 42 °C and grown in Luria Broth (LB) at 37 °C. *E. coli* cells were induced with 0.5 mM IPTG to induct protein expression. Cells were harvested by centrifugation at 5000 × *g*, 4 °C for 15 min and the pellet was resuspended in lysis buffer (20 mM Tris pH 7.9, 500 mM NaCl, 5 mM imidazole, 2 mM DTT and 1x protease inhibitor cocktail). Cells were then sonicated and centrifuged at 14,000 × *g*, 4 °C for 15 min. 1 ml of Ni-NTA was added to the supernatant and incubated at 4 °C with end-to-end mixing for 2 h and subjected to a column. The Ni-NTA resin was washed with 10 ml wash buffer (20 mM Tris pH 7.9, 250 mM NaCl, 15 mM Imidazole and 0.5 mM DTT) and eluted with 1 ml elution buffer (20 mM Tris pH 7.9, 250 mM NaCl and 1 M imidazole). The elute was then concentrated by using a protein concentrator (Thermo Scientific™ Pierce™) and resuspend in dialysis buffer (50 mM Tris pH 7.9, 50 mM NaCl, 2 mM DTT, and 10% glycerol). The purified protein was stored at −80 °C.

pGEX-4T1-SENP1 and its mutant were purified using fowllowing buffers: lysis buffer (1 mM DTT, and 1x protease inhibitor cocktail, 800 mM NaCl, 5 mM EDTA, 50 mM Tris-pH8.0, 0.1% Triton x-100), wash buffer (800 mM NaCl, 5 mM EDTA, 50 mM Tris-pH8.0, 0.1% Triton x-100), elution buffer (40 mM glutathione, 800 mM NaCl, 5 mM EDTA, 50 mM Tris-pH8.0, 0.1% Triton x-100), dialysis buffer (50 mM Tris pH 7.9, 50 mM NaCl, 2 mM DTT, and 10% glycerol)^36,37^.

### RNA interference

siRNA transfections were performed with 100 nm siRNA oligonucleotide duplexes using Lipofectamine^TM^ RNAiMAX (Invitrogen) according to the manufacturer’s instructions. Cells were harvested for analysis 48 h after transfection. siRNA oligonucleotides against SENP1 were purchased to target non-coding sequences (cat. no. D-006357-01-0002, D-006357-01-0002, Dharmacon). Two siRNAs were obtained similar knockdown efficiency. Negative control GL2: 5ʹ-AACGTACGCGGAATACTTCGA dTdT-3ʹ.

### Immunofluorescence microscopy

Cells grown on coverslips in 6-well plates were transfected with siRNA or plasmid for 48 h. After fixation with 4% paraformaldehyde in PBS for 10 min, cells were penetrated with 0.05% Triton x-100 in PBS for 5 min. Cells were then blocked with blocking buffer (3% BSA in 0.02% Triton x-100 PBS) for 30 min, followed by incubation with primary antibody overnight. The antibody was then washed with washing buffer (0.02% Triton x-100 in PBS) 3×5 min, followed by incubation with 512nm-conjugated secondary antibody for 45 min. After washing, the cells were counterstained with 15 μl of 10 ng/ml DAPI and the fluorescence was visualized by Zeiss Axioskop fluorescence microscope and analyzed by Zeiss AxioVision deconvolution software.

### Cell viability assay

Cells were seeded at 3000 cells/well in 96-well plates were treated with drugs, followed by incubation for 48 h, after which cell viability was detected using Sulforhodamine B (SRB) assay^38^. Absorbance at 510 nm was detected using a SpectraMax Reader (Molecular Devices) and analyzed with SoftMax Analysis Software (Molecular Devices). Combinational index (CI) values were calculated using CompuSyn software^39^.

### Clonogenic assay

Cells seeded in 6-well plates were treated with cisplatin, Mc or an equivalent volume of DMSO for 48 h. Thereafter, the cells were incubated in drug free media for 14 days. Colonies were visualized by crystal violet staining, imaged with an EPSON scanner with Photoshop software and county using Quantity One® software.

### Genome-wide RNA-sequencing (RNA-Seq)

The assay was previously described^4^. Briefly, cells growing in log phase were harvested and RNA isolation was completed using the Qiagen miRNeasy Mini Kit. RNA was then converted into cDNA libraries using the Illumina TruSeq Stranded mRNA sample preparation kit (Illumina # RS-122-2103). Read counts of each sample were normalized with DESeq and ran against their negative binomial two sample test to find significant genes that higher transcript abundance in either the IGROV1 or IGROV1 CR. Genes with false discovery rate (FDR) < 0.05, fold change larger than 2 or smaller than 0.5-fold were considered as differentially expressed genes.

### Animal experiments

Xenograft experiments were performed in 6-week female BALB/c athymic nude mice (Jackson Laboratory) by subcutaneously injecting 5 × 10^6^ IGROV1 cells within 50% Matrigel gelatinous protein mixture (Corning). Mice were randomized to receive treatment after reached a minimum tumor volume of 150 mm^3^. 3 groups of mice (5 mice per group) were treated intraperitoneally with vehicle (Control), carboplatin (20 mg/kg/2day) and carboplatin (60 mg/kg/2day) for 2 weeks. The investigators were not blinded to the group allocation and data collection. All animal experiments were conducted in accordance with the Institution Animal Care and Use Committee of the George Washington University for laboratory animal use and care, and all relevant ethical regulations were followed.

### RT-qPCR

The assay was performed exactly as previously described^4^. The expression level of SENP1 was normalized to GAPDH and the results were given as relative copy numbers. Subsequently, the expression level of SENP1 in cisplatin-resistant cells was normalized to that of parental cells.

For samples from patients, the expression levels of SENP1 in 61 platinum-sensitive and 32 platinum-resistant patients were measured using GAPDH as internal control. Subsequently, the expression level of SENP1 in platinum-resistant patient samples was normalized to the mean copy number of SENP1 in platinum-sensitive patient samples. All studies were approved by the local ethics committees (institutional review board reference No: UW 05-143 T/806 and UW 11-298) and the studies abide by the Declaration of Helsinki principles. Informed consent was received before inclusion in the study.

### Statistical analysis

GraphPad Prism 5.0 software was used for statistical analysis. Data were represented as the mean ± S.D. Statistical analysis was performed using one-way ANOVA or Student’s *t* test. *P* < 0.05 was considered significant. For Kaplan–Meier survival analysis, a Log-rank (Mantel-Cox) test was used to compare each of the arms.

## Results

### SENP1 directly interacts with and deSUMOylates JAK2

**Fig. 1:**
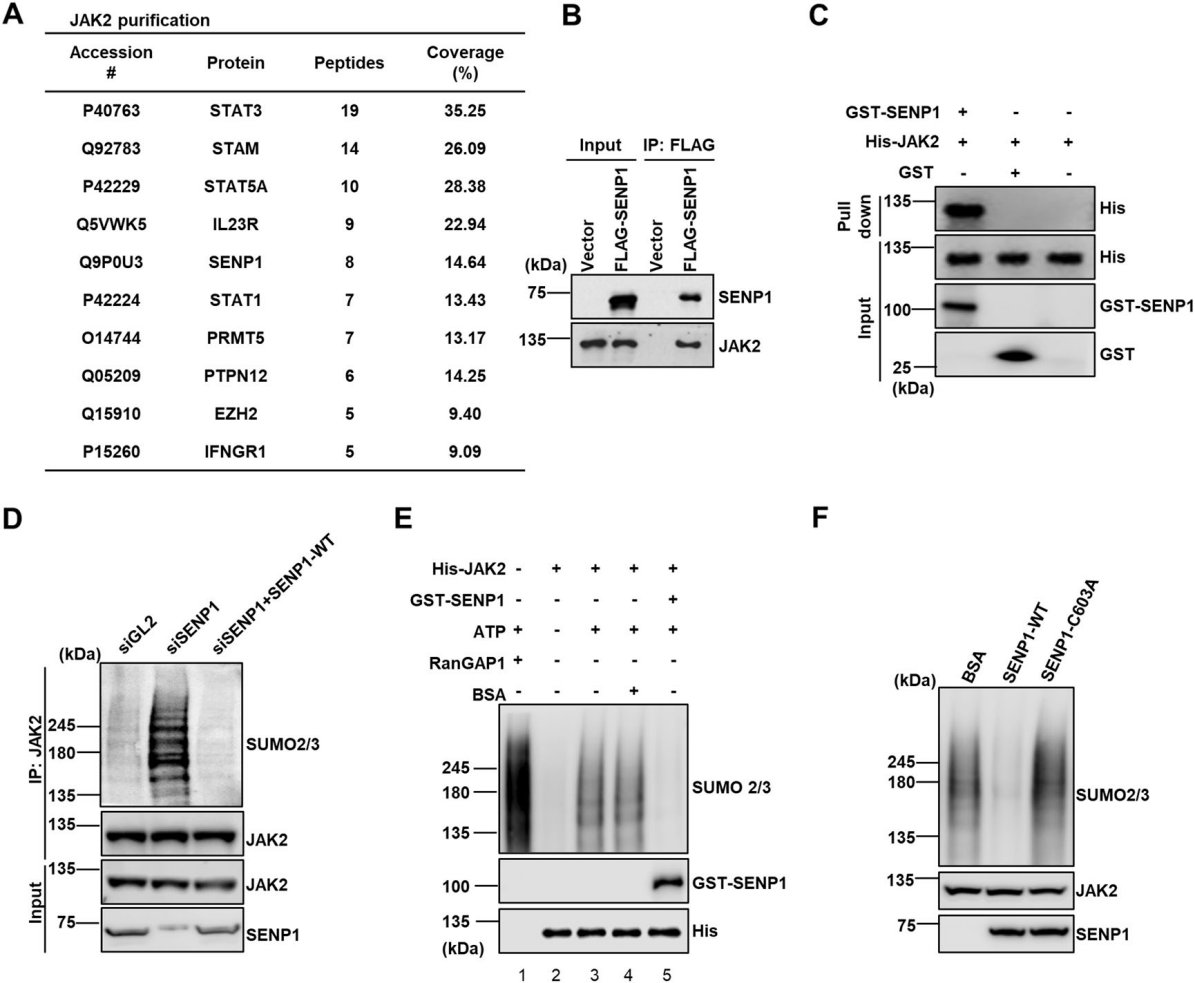
Identification of SENP1 that directly interacts with and deSUMOylates JAK2. **A** Mass spectrometry analysis to identify JAK2 associated proteins. Immunoprecipitation of His-tagged JAK2 from HEK293T cells was used for mass-spec analysis. Results represent the proteins identified in cells expressing His-hJAK2 compared cells expressing empty vector. Peptides were filtered to exclude false positives. **B** SENP1 interacts with JAK2 in vivo. Co-IPs of Flag-SENP1 were analyzed by Western blot using indicated antibodies. **C** SENP1 interacts with JAK2 in vitro. Recombinant GST-SENP1 was used to capture His-JAK2 protein on glutathione agarose as described in “Materials and Methods”. **D** Ectopic expression of SENP1-WT restores JAK2 deSUMOylation in SENP1 knockdown IGROV1 cells. SUMOylated JAK2 was captured by using IP with anti-JAK2 antibody, followed by Western blot using antibodies against SUMO2/3. **E** SENP1 deSUMOylates JAK2 in vitro. Recombinant GST-SENP1 and His-JAK2 were used in SUMOylation assay as described in “Materials and Methods”. **F** SENP1-C603A fails to deSUMOylate JAK2 in vitro. Recombinant GST-SENP1, GST-SENP1-C603A and His-JAK2 were used in SUMOylation assay as described in “Materials and Methods”.

To identify proteins that regulate JAK2 activity, we purified JAK2 proteins using HEK293T cells expressing His-tagged JAK2. The immunoprecipitated His-JAK2 and its associated proteins were subjected to mass spectrometry analysis that identified a number of well-known JAK2-associated proteins, including STAT3, STAT5A, and STAT1. Interestingly, we also identified SENP1 from JAK2 immunoprecipitates (Fig. 1A). To validate the SENP1-JAK2 interaction in cells, we expressed FLAG-tagged SENP1 (FLAG-SENP1) in HEK293T cells followed by immunoprecipitation with FLAG antibody. As shown in Fig. 1B, JAK2 was detected from IPs of FLAG-SENP1. To determine whether SENP1 and JAK2 interact directly, we performed in vitro affinity capture assay by using purified recombinant glutathione-S-transferase-tagged-SENP1 (GST-SENP1) to pull down His-tagged-JAK2 (His-JAK2). While GST alone did not capture His-JAK2, GST-SENP1 successfully pulled down His-JAK2, indicating a direct interaction between SENP1 and JAK2 (Fig. 1C).

To determine the functional consequence of SENP1-JAK2 interaction, we first examined whether inhibition of SENP1 affects SUMOylation of JAK2. To this end, we immunoprecipitated JAK2 and evaluated the SUMOylation level of JAK2 using antibody against SUMO2/3 in SENP1 depleted cells. As shown in Fig. 1D, SENP1 knockdown resulted in the significant increase of JAK2 SUMOylation. To rule out the off-target effect, we ectopically expressed siRNA resistant FLAG-SENP1-WT in SENP1 depleted IGROV1 cells and found that expression of SENP1-WT significantly restored the deSUMOylation of JAK2 in SENP1 knockdown cells (Fig. 1D).

To further determine whether SENP1 can directly deSUMOylate JAK2, we performed in vitro deSUMOylation assays by using purified recombinant His-JAK2 and GST-SENP1 proteins. Using a SUMOylation kit (ENZO Life Sciences, Catalog # BML-UW8955), the positive control protein RanGAP1 was found to be SUMOylated (Fig. 1E, lane 1). Like RanGAP1, JAK2 was also found to be SUMOylated as indicated by multiple upward shifts in the apparent molecular mass of the protein (Fig. 1E, lane 3). Significantly, incubation of GST-SENP1 but not BSA with JAK2 after JAK2 SUMOylation reaction completely removed SUMO from JAK2 in vitro (Fig. 1E, lane 5). Consistently, purified catalytically inactive GST-SENP1-C603A proteins exhibited lower activity to deSUMOylate JAK2 compared to GST-SENP1-WT in vitro (Fig. 1F), indicating deSUMOylation of JAK2 by SENP1 requires SENP1 protease activity. Taken together, our data suggest that SENP1 directly interacts with and deSUMOylates JAK2 both in vitro and in vivo.

### DeSUMOylation of JAK2 by SENP1 promotes cytoplasmic accumulation of JAK2

**Fig. 2:**
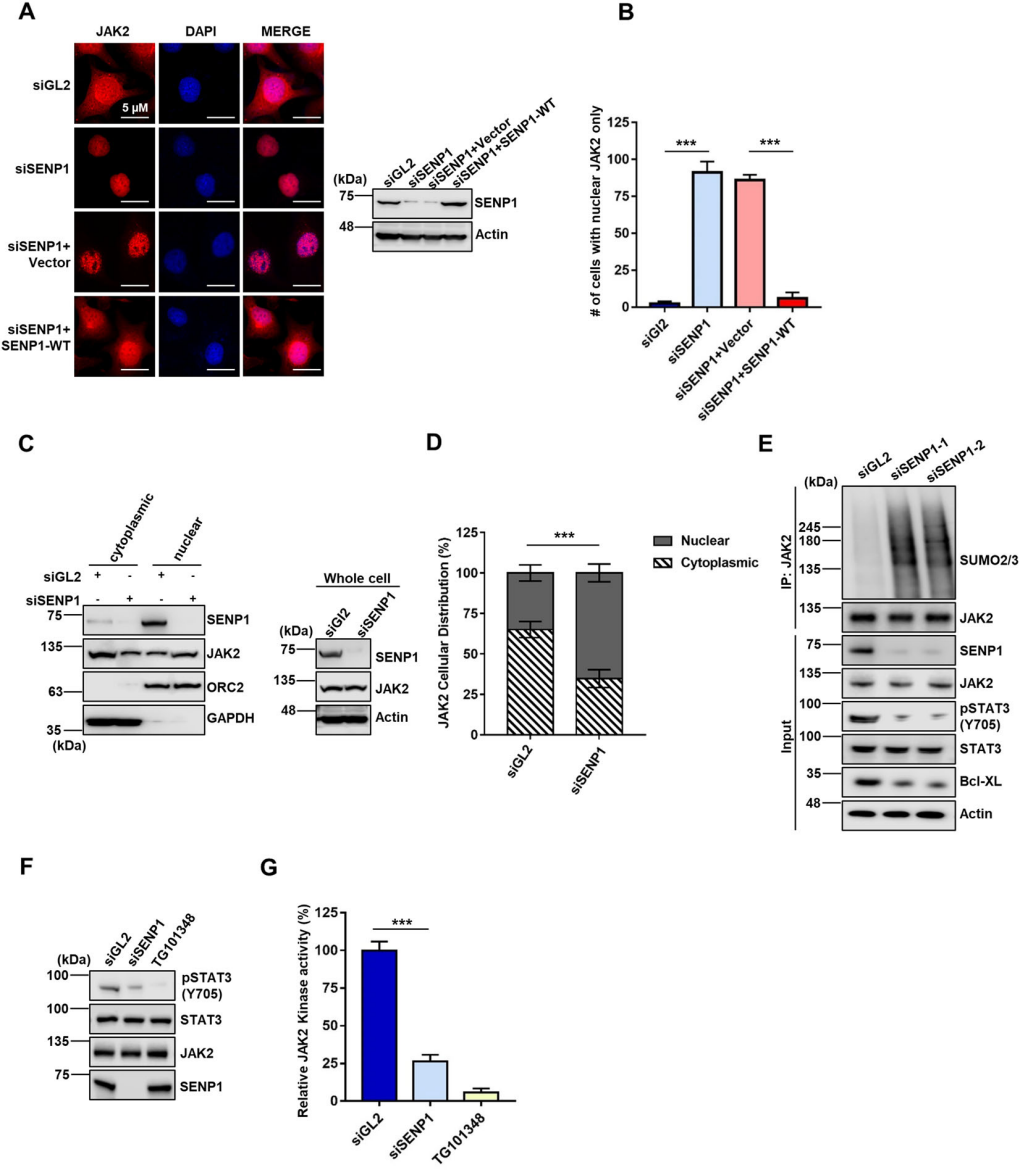
DeSUMOylation of JAK2 by SENP1 promotes cytoplasmic accumulation of JAK2. **A** Cellular distribution of JAK2 in IGROV1 cells treated with siSENP1 or siSENP1 with ectopic expression of siRNA resistant SENP1. JAK2 localization was detected by immunofluorescence assay using anti-JAK2 antibody. Scale bar: 5 µm. Right panel, SENP1 expression in treated cells. **B** Quantification of JAK2 cellular distribution in IGROV1 cells as shown in (**A**). In total, 100 cells were counted in each sample. ***, *p* < 0.001. **C** Nuclear fractionation of JAK2 in IGROV1 cells treated with siGL2 or siSENP1, followed by Western blot for indicated proteins. **D** Quantification of JAK2 fractionations shown in (**C**). ***, *p* < 0.001. **E** SENP1 knockdown decreases expression of pSTAT3 (Tyr705) and Bcl-Xl. IGROV1 cells treated with indicated siRNAs, followed by Western blot with indicated proteins. **F** SENP1 knockdown decreases pSTAT3 (Tyr705) by JAK2 in vitro. Recombinant STAT3 was incubated with purified JAK2 from IGROV1 cells treated with siRNAs or JAK2 inhibitor (TG101348) for in vitro kinase assay as described in “Materials and Methods”. Cells lysates were resolved in SDS-PAGE gel, followed by Western blot for indicated proteins. **G** Quantification of pSTAT3 (Tyr705) levels in three independent experiments. ***, *p* < 0.001.

Cytoplasmic and nuclear JAK2 proteins have distinct cellular functions^17^. Since JAK2 SUMOylation has been reported to regulate the translocation of JAK2 between nucleus and cytoplasm with unknown regulatory mechanism^40^, we therefore hypothesized that deSUMOylation of JAK2 by SENP1 controls JAK2 localization and thus affecting JAK2 activity. To test this hypothesis, we visualized endogenous JAK2 in IGROV1 cells with downregulation of SENP1 by siRNA. In control siGL2 treated cells, JAK2 localized in both cytoplasm and nucleus, whereas depletion of SENP1 by siRNAs led to the accumulation of JAK2 in nucleus (Fig. 2A and B). Ectopic expression of SENP1 restored the localization of JAK2 in cytoplasm (Fig. 2A and B). Consistently, the nuclear fractionation assay in IGROV1 cells revealed that nuclear JAK2 was significantly increased while cytoplasmic JAK2 was decreased in SENP1 knockdown cells as compared to siGL2 treated cells (Fig. 2C and D).

Phosphorylation of STAT3 at tyrosine 705 (Y705) by JAK2 is one of the most important and well established cytoplasmic functions of JAK2^41,42^. To determine whether accumulation of JAK2 in nucleus by depleting SENP1 affects JAK2 cytoplasmic activity, we evaluated phosphorylated STAT3 (pSTAT3 (Y705)) level in SENP1 depleted IGROV1 cells. Compared to siGL2, cells treated with two independent siSENP1s exhibited the significant decrease of pSTAT3 and anti-apoptotic protein Bcl-xL (Fig. 2E). To further confirm that SENP1 regulates JAK2 activity, we performed cell-free kinase assay using JAK2 purified from IGROV1 cells treated with siSENP1 or JAK2 inhibitor TG101348. Consistently, purified JAK2 from cells treated with siSENP1 or TG101348 showed the decrease of phosphorylation of recombinant substrate STAT3 proteins (Fig. 2F and G), indicating that the activity of JAK2 is significantly reduced by SENP1 depletion or JAK2 inhibitor. Taken together, our data suggest that SENP1controls JAK2 activity by regulating its cellular localization in cells.

### JAK2 SUMOylation is required for STAT3 phosphorylation

**Fig. 3:**
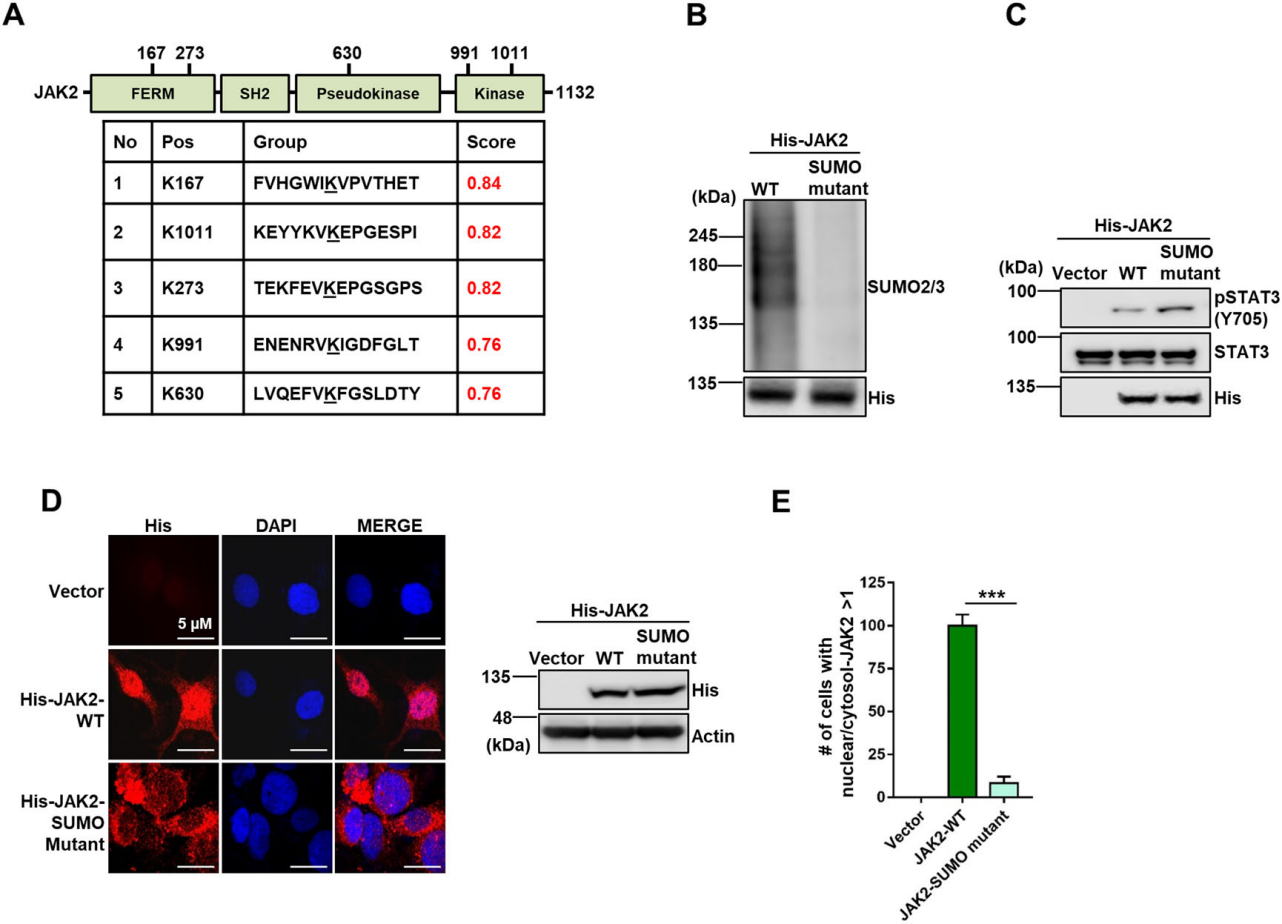
JAK2 SUMOylation is required for STAT3 phosphorylation. **A** Schematic diagram of the distribution of putative JAK2 SUMOylation sites, predicted by SUMOplot™. The score indicates the probability. **B** JAK2-SUMO mutant clones 1 and 2 fails to be SUMOylated in vitro. Recombinant His-JAK2 proteins were used for in vitro SUMOylation assay as described in “Materials and Methods”. **C** JAK2-SUMO mutant phosphorylates STAT3 (Tyr705) in vitro. Empty vector, His-JAK2 and His-JAK2 SUMO mutant were purified from HEK293T cells, followed by in vitro kinase assay as in Fig. 2F. **D** JAK2-SUMO mutant accumulates in cytoplasm. Empty vector, His-JAK2 and His-JAK2 SUMO mutant were transfected in IGROV1 cells, followed by immunofluorescence assay using anti-His antibody. Scale bar: 5 µm. Right panel, expression of JAK2 and JAK2-SUMO mutant. **E** Quantification of cellular distribution of JAK2 in IGROV1 cells as shown in (**D**). In total, 100 cells were counted in each sample. ***, *p* < 0.001.

To further determine the function of JAK2 SUMOylation, we identified 5 potential SUMOylation sites of JAK2 by using SUMOplot™ prediction program (http://www.abgent.com/tools). Four of these sites have been reported previously (K167, K630, K991, and K1011), but mutations of one or two of these sites did not significantly abolish JAK2 SUMOylation^40^. We therefore mutated all 5 lysine residues to alanine (JAK2-K167A-K273A-K630A-K991A-K1011A) to create a mutant JAK2 (JAK2-SUMO mutant) (Fig. 3A). The cell-free SUMOylation assay indicated that JAK2-SUMO mutant failed to be SUMOylated as compared to JAK2-WT (Fig. 3B). Moreover, the kinase assay indicated that JAK2-SUMO exhibited the increased kinase activity on recombinant STAT3 (Fig. 3C). Given that SENP1 depletion decreased cytoplasmic activity of JAK2, we assumed that JAK2-SUMO mutant should be sequestered in cytoplasm. Indeed, immunofluorescence staining in IGROV1 cells showed that the JAK2-SUMO mutant, unlike wild type JAK2, was accumulated in cytoplasm (Fig. 3D and E). Together, these results suggest that SENP1 regulates JAK2 activity via deSUMOylation.

### SENP1 is upregulated in cisplatin resistant cancer cells

**Fig. 4:**
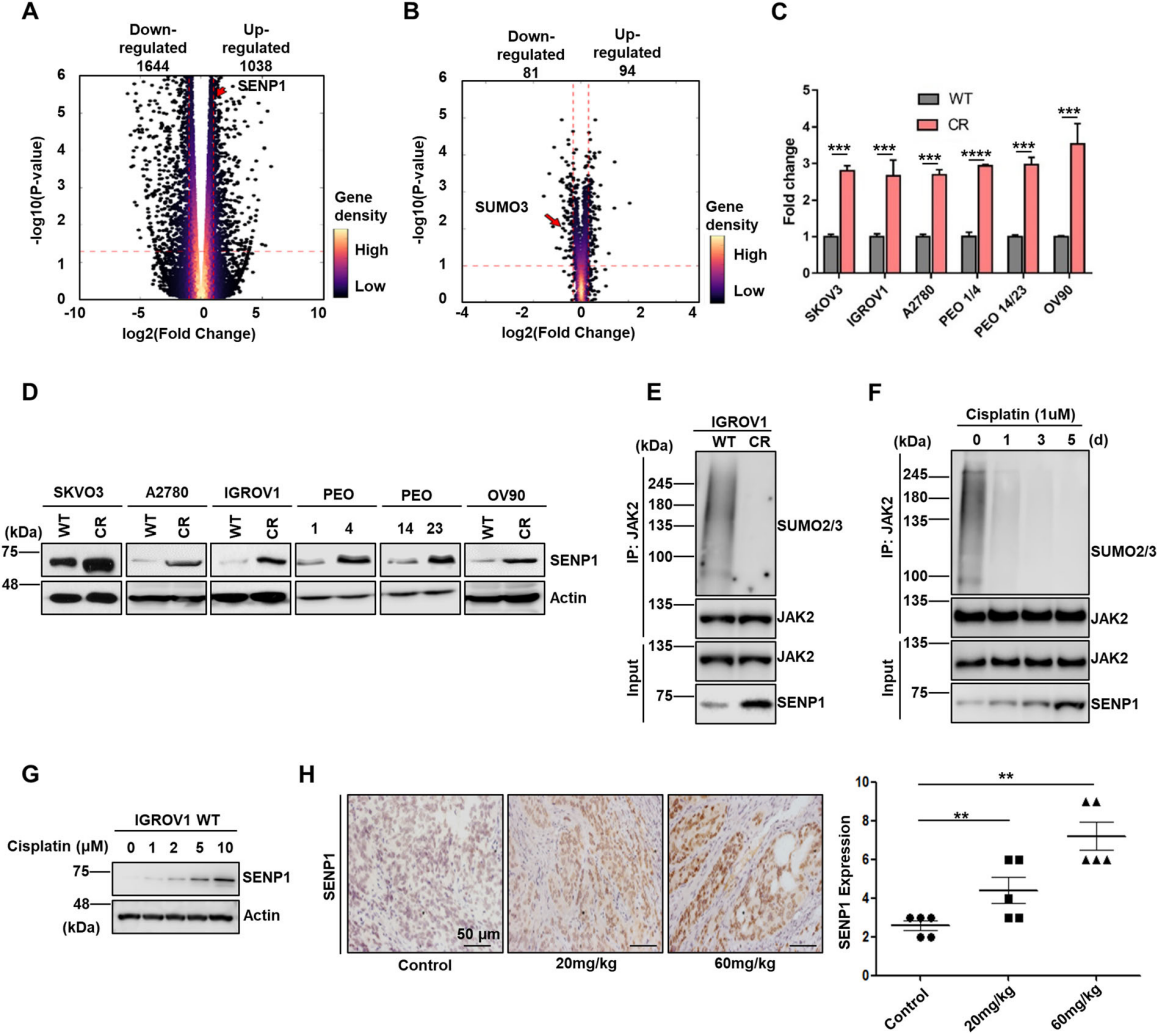
SENP1 is upregulated in cisplatin resistant cancer cells. **A** Volcano plot of up-regulated and down-regulated genes identified from RNA-Seq assay using IGROV1 and IGROV1 CR cells. Genes are ranked in volcano plot according to their p-value and relative abundance ratio (log2 fold change). **B** Volcano plot of up-regulated and down-regulated genes identified by quantitative proteomics using IGROV1 and IGROV1 CR cells. Proteins are ranked in volcano plot according to their p-value and their relative abundance ratio (log2 fold change). **C** SENP1 mRNA expression levels are significantly upregulated in cisplatin resistant ovarian cancer cell lines. mRNA expression levels were assessed by qPCR. **D** SENP1 protein levels are upregulated in indicated paired cisplatin sensitive and resistant ovarian cancer cell lines. Cell lysates from indicated cells were Western blotted for indicated proteins. ***, *p* < 0.001; ****, *p* < 0.0001. **E** SUMOylation of JAK2 decreases in cisplatin-resistant IGROV1 cells. Cell lysates from IGROV1 and IGROV1 CR cells were Western blotted for indicated proteins. **F** SUMOylation of JAK2 decreases in IGROV1 cells treated with cisplatin. Cell lysates of IGROV1 CR cells treated with cisplatin at indicated time point were Western blotted for indicated proteins. **G** IGROV1 cells were treated with indicated amount of cisplatin, followed by Western blot for indicated proteins. **H** Representative IHC images of IGROV1 xenograft tumors treated with vehicle and carboplatin (20 mg/kg and 60 mg/kg). Scale bar: 50 µm. Right panel, Quantification of IHC staining for SENP1 in carboplatin treated mice. *n* = 5 mice/group. Data were represented as means ± SD (*n* = 3). **, *p* < 0.01.

In an attempt to identify genes that regulate platinum drug resistance in ovarian cancer cells, we conducted both RNA-Seq and quantitative mass-spec analyses. These assays found that SENP1 mRNA was highly upregulated (Fig. 4A), while SUMO3 level was decreased in platinum-resistant ovarian cancer cells (IGROV1 CR) (Fig. 4B), suggesting a possible role of SENP1 in the regulation of platinum-resistance via SUMOylation in ovarian cancer. We have reported that activated JAK2 is critical for platinum-resistance in ovarian cancer cells^4^, we therefore hypothesized that SENP1/JAK2 link plays an important role in the regulation of platinum-resistance. To test this hypothesis, we examined the expression level of SENP1 mRNA in six paired sensitive an resistant ovarian cancer cell lines, including SKOV3 WT, SKOV3 CR, IGROV1 WT, IGROV1 CR, A2780 WT, A2780 CR, PEO1, PEO4, PEO14, PEO23, OV90 WT and OV90 CR. Significantly, all cisplatin resistant cells exhibited 2.8-4-fold increase of SENP1 mRNA levels compared to their sensitive counterparts (Fig. 4C). Consistently, the protein levels of SENP1 were also upregulated in platinum-resistant ovarian cancer cells, in which SUMOylation of JAK2 was decreased correspondingly (Fig. 4D and E).

If SENP1/JAK2 regulates platinum-resistance, it is likely that SENP1/JAK2 axis is activated in response to platinum drug treatment. Indeed, SENP1 protein level was increased, while JAK2 SUMOylation was decreased correspondingly in IGROV1 cells treated with cisplatin in a time and dose dependent manner (Fig. 4F and G). To further test the SENP1 induction by platinum drugs in vivo, we measured SENP1 protein levels in IGROV1 xenograft tumors by using immune histochemistry (IHC) in implanted immunodeficient nude mice that were treated with carboplatin at 20 mg/kg or 60 mg/kg after tumor was developed. As expected, carboplatin treated ovarian tumors exhibited a significant increase of SENP1 expression (Fig. 4H). These results confirmed that SENP1 expression is increased in response to platinum drug treatment in ovarian cancer cells.

### SENP1 is upregulated in a manner dependent on RUNX2 in cisplatin resistant cancer cells

**Fig. 5:**
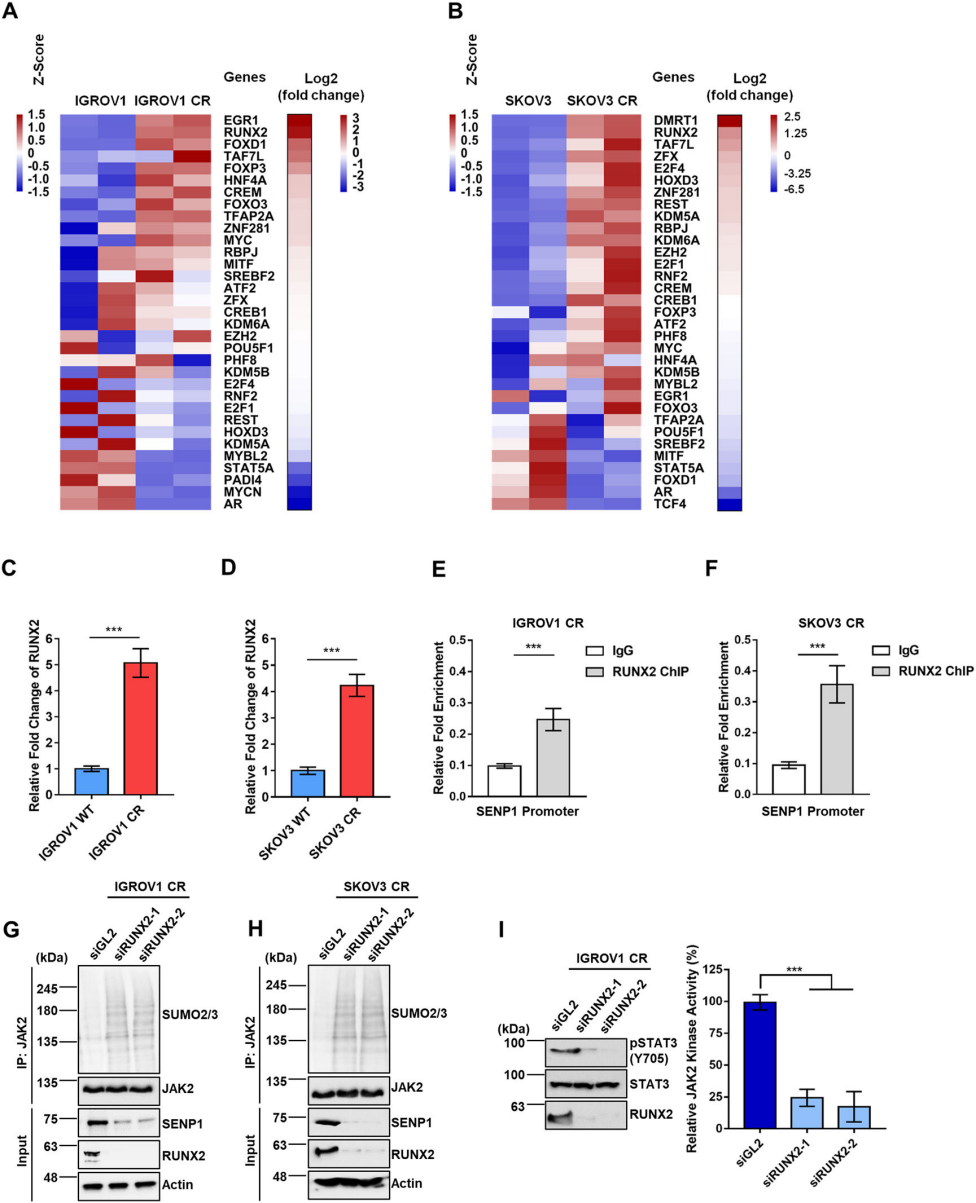
Transcription factor RUNX2 regulates SENP1 expression in cisplatin-resistant ovarian cancer cells. **A** Heat map of transcription factors of SENP1 in IGROV1 CR/ IGROV1 cells and (**B**) SKOV3 CR/SKOV3 cells. Transcriptional factors were identified from database (http://amp.pharm.mssm.edu/Harmonizome/gene/SENP1) and matched with their mRNA expression values by RNA-Seq analyses. Colors in the heat map represent the mRNA expression levels after z-score normalization across different samples. **C** SENP1 mRNA expression levels are significantly downregulated in IGROV1 CR and (**D**) SKOV3 CR cells treated with siRUNX2. mRNA expression levels were assessed by qPCR. ***, *p* < 0.001. **E** ChIP assay to detect the association of RUNX2 with promoter regions of SENP1 in IGROV1 CR cells and (**F**). SKOV3 CR cells. Cells were harvested and cell lyses were immunoprecipitated against IgG and anti-RUNX2 antibodies. RUNX2-associated DNA were examined by qPCR. Data were represented as mean ± SD (*n* = 3). ***, *p* < 0.001. **G** SENP1 protein expression levels are downregulated in IGROV1 CR and (**H**). SKOV3 CR cells treated with siRUNX2. **I** RUNX2 knockdown decreases pSTAT3 (Tyr705) by JAK2 in vitro. Recombinant STAT3 was incubated with purified JAK2 from IGROV1 CR cells treated with siGL2, siRUNX2-1, or siRUNX2-2 for in vitro kinase assay as described in “Materials and Methods”. Right panel, Quantification of pSTAT3 (Tyr705) levels in three independent experiments. ***, *p* < 0.001.

Having found that SENP1 mRNA level is upregulated in cisplatin resistant cell lines as compared to that in their sensitive counterparts (Fig. 4C), we next investigated how SENP1 mRNA is regulated. To this end, we first identified all transcription factors (TF) that target promotor region of SENP1 from the Harmonize database (http://amp.pharm.mssm.edu/Harmonizome/gene/SENP1). Comparing the mRNA level of these TFs with RNA sequencing data (Fig. 5A and B), we identified RUNX2, whose mRNA levels were significantly increased in both resistant cell lines. qPCR analyses indicated that RUNX2 mRNA levels were indeed increased significantly in resistant IGROV1 CR and SKOV3 CR cells compared to sensitive cells (Fig. 5C and D). To explore whether RUNX2 binds to SENP1 promoter region in ovarian cancer cells, we performed ChIP assay and found that RUNX2 was enriched at promotor region of SENP1 but not at a negative region of SENP1 promotor, indicating that RUNX2 directly binds to SENP1 promotor in both IGROV1 CR and SKOV3 CR cells (Fig. 5E and F). We next tested whether RUNX2 regulates SENP1/JAK2 axis. As shown in Fig. 5G and H, depletion of RUNX2 reduced SENP1 expression and increased SUMOylated JAK2 in IGROV1 CR cells and SKOV3 CR cells. Consistent with JAK2 activity in cells treated with siSENP1 (Fig. 2F), purified JAK2 from cells treated with siRUNX2 showed decreased phosphorylation of recombinant substrate STAT3 proteins (Fig. 5I). Together, these results suggest that RUNX2 is a critical transcription factor regulating SENP1 expression in platinum-resistant ovarian cancer.

### Clinical evidence of activated SENP1/JAK2 signaling in platinum-resistant ovarian cancer patients

**Fig. 6:**
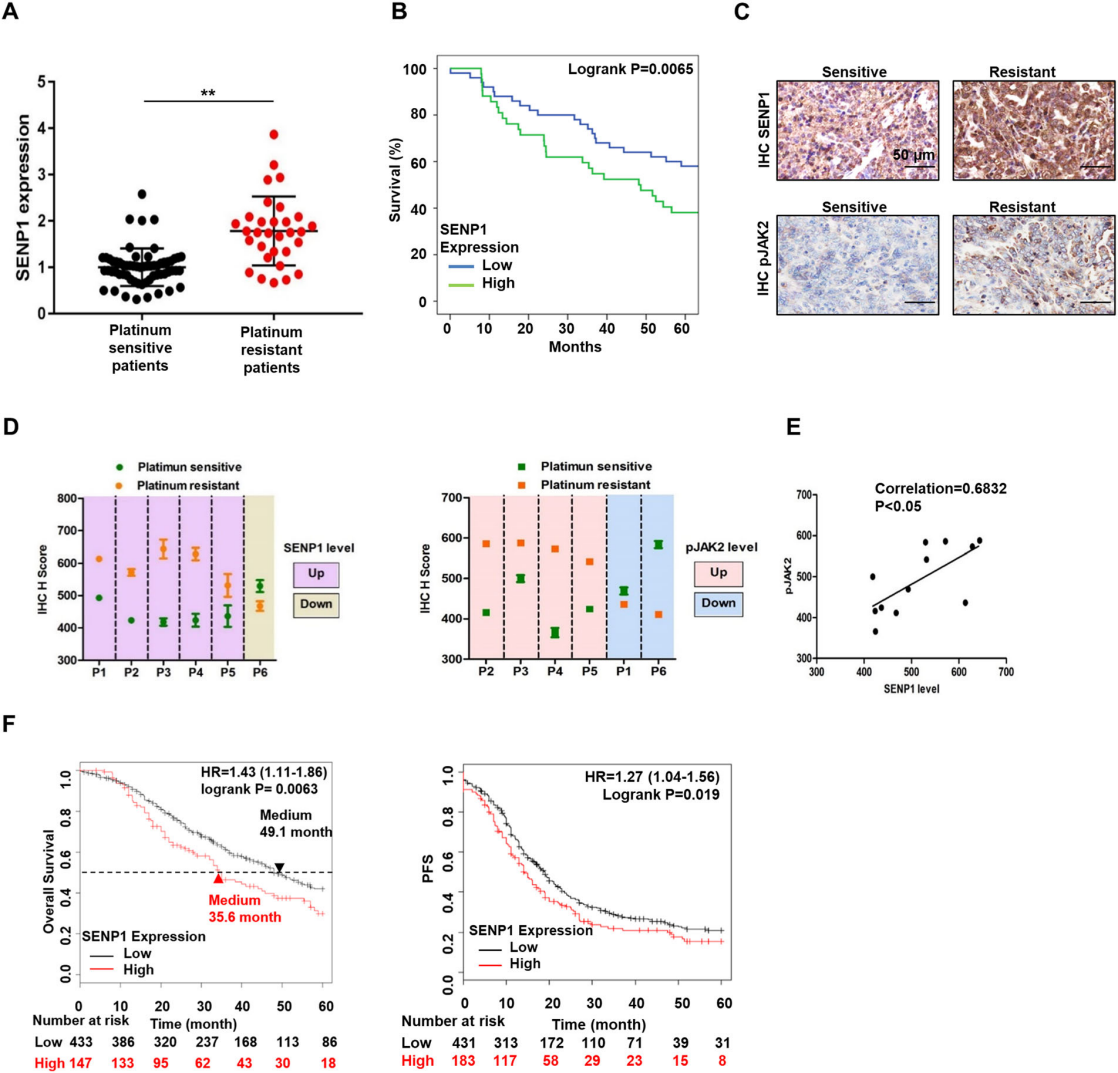
Clinical evidence of activated SENP1/JAK2 activity in platinum-resistant ovarian cancer patients. **A** SENP1 mRNA expression level in platinum-sensitive and -resistant ovarian cancer patients. Number of patients evaluated: 61 (platinum-sensitive), 32 (platinum-resistant). mRNA expression levels were assessed by qPCR. **, *p* < 0.01. **B** Kaplan–Meier analyses of 5-year overall survival rates of patients as shown in (**A**). **C** Representative SENP1 IHC staining from the same patient before and after development of chemoresistance. **D** Quantification of IHC scores from 6 patients. Left, SENP1 IHC, Right, phosphorylated JAK2. Data are represented as mean ± SD. **E** Correlation of IHC scores between SENP1 and pJAK2 expression in samples from six patients. **F** Kaplan–Meier analyses of 5-year overall survival rates (left), and progression-free survival rates (right) based on clinical and molecular data from ovarian cancer patients (OS: *n* = 1581; PFS: *n* = 1435)^29^. Patients were stratified by SENP1 mRNA expression in their tumors. Medium survival, log-rank (Mantel-Cox) *p* values and hazard ratios (HR). 95% confidence interval in parentheses were shown.

To further confirm the role of SENP1 in platinum-resistance of ovarian cancer patients, we measured SENP1 mRNA expression levels from tumor samples of 61 platinum-sensitive patients and 32 platinum-resistant patients. SENP1 mRNA levels were significantly increased in platinum-resistant patients (Fig. 6A). Significantly, the patients with higher SENP1 mRNA expression level exhibited worse prognosis in terms of overall survival as compared to the patients with lower SENP1 mRNA level (Fig. 6B). To directly evaluate protein expression change caused by chemotherapy, we also compared SENP1 protein expression levels in the samples from the same patient before platinum drug treatment and after acquired drug resistance. Strikingly, 5 out of 6 patients showed upregulated SENP1 protein level after development of platinum drug resistance (Fig. 6C and D). There was a good correlation between SENP1 and phosphorylated JAK2 levels in these tested sensitive and resistant patient tumor samples (Fig. 6E), indicating SENP1 upregulation is correlated with JAK2-mediated platinum-resistance in ovarian cancer.

We next analyzed the correlation of survival rate with the SENP1 expression from patients who had platinum drug treatment history from ovarian cancer databases (http://kmplot.com/analysis/index.php?p=service&cancer=ovar). We found that patients with high expression level of SENP1 exhibited a poor prognosis of overall survival and progression free survival (Fig. 6F). Thus, a higher level of SENP1 is highly correlated with worse ovarian cancer patient survival following platinum drug-based therapy.

### Overcoming cisplatin resistance of ovarian cancer by inhibiting SENP1

**Fig. 7:**
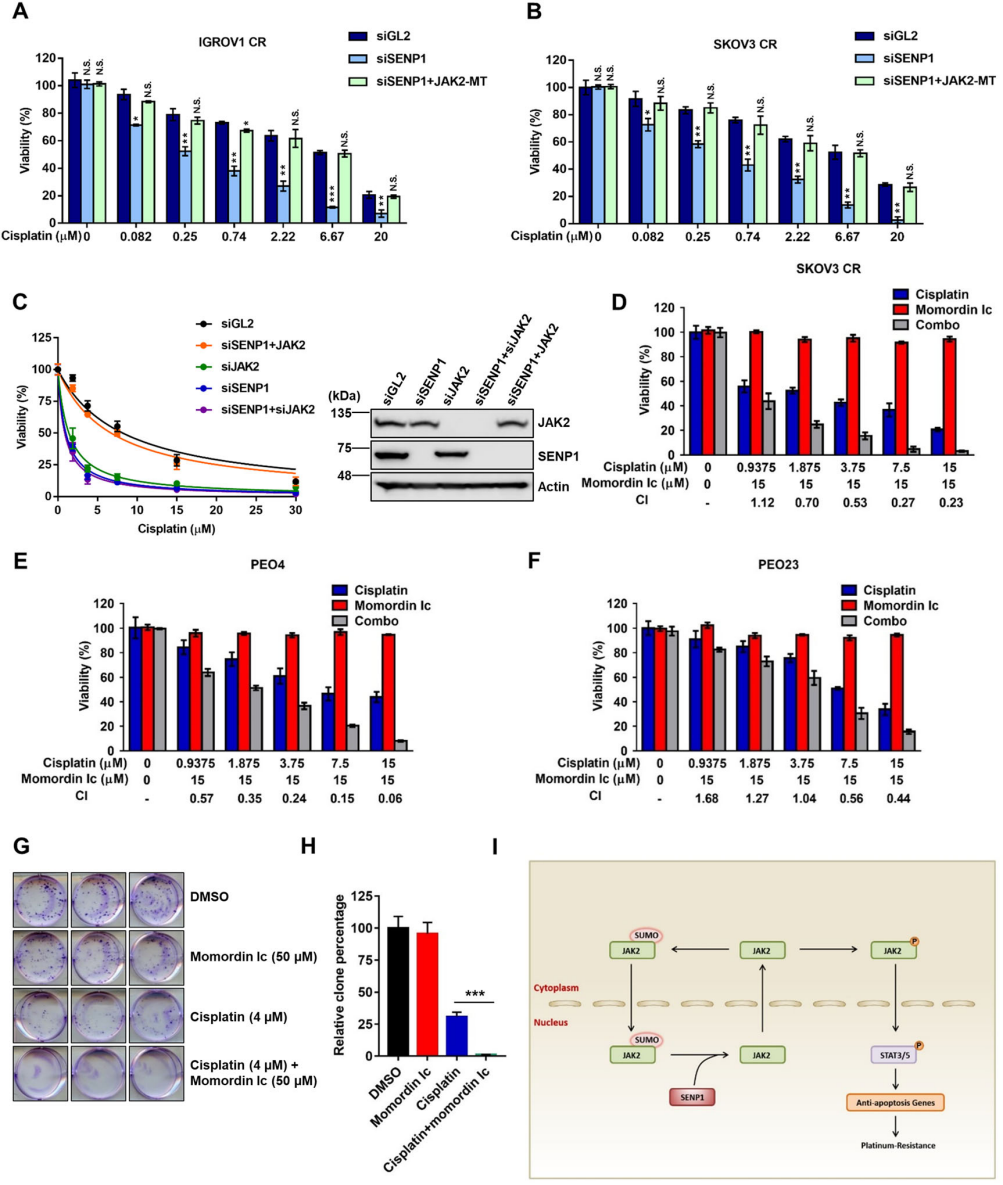
SENP1 is a potential target for overcoming cisplatin resistance. **A** Cell viability of IGROV1 CR treated with indicated siRNAs and JAK2. Data are represented as mean ± SD (*n* = 3). N.S., not significant; *, *p* < 0.05; **, *p* < 0.01; ***, *p* < 0.001. **B** SKOV3 CR cells treated with indicated siRNAs and JAK2. Data are represented as mean ± SD (*n* = 3). N.S., not significant; *, *p* < 0.05**;** **, *p* < 0.01. siSENP1 + JAK2-MT, ectopic expression of His-JAK2-SUMO mutant in SENP1 knocked down cells. **C** Ectopic expression of JAK2 restores cisplatin resistance in SENP1 depleted cells. IGROV1 CR cells treated with indicated siRNAs were transfected with vector JAK2 plasmids. Cell viability was analyzed by using cell viability assay as described in “Materials and Methods”. Right, the expression of indicated proteins in cells treated in (**C**). **D** The synergistic effects of cisplatin and Momordin Ic in SKOV3 CR, (**E**). PEO4 and (**F**). PEO23 cells. Concentrations of cisplatin and Momordin Ic as well as the CI index were indicated. Data are represented as mean ± SD (*n* = 3). **G** Representative colony formation and (**H**) quantification of IGROV1 CR cells treated with cisplatin and Momordin Ic. Colonies were stained with crystal violet. Data are represented as mean ± SD (*n* = 3). ***, *p* < 0.001. **I** Working model of SENP1-mediated deSUMOylation of JAK2 regulates its kinase activity and platinum drug resistance.

To test whether SENP1 contributes to cisplatin-resistance in ovarian cancer cells, we depleted SENP1 by siRNA and found SENP1 depletion significantly increased the cell sensitivity to cisplatin in both SKOV3 CR and IGROV1 CR cells (Fig. 7A and B). Ectopic expression of His-JAK2-SUMO mutant (MT) restored cell survival of cells with downregulation of SENP1 (Fig. 7A and B). Given that SENP1 has other targets^26–28,43,44^, it is possible that SENP1 may promote platinum-resistance by regulating other proteins rather than JAK2. To confirm that JAK2 is the primary target of SENP1 to promote platinum-resistance, we examined the sensitivity of IGROV1 CR cells to cisplatin using genetic analyses. As shown in Fig. 7C, cells with depletion of either SENP1 or JAK2 exhibited the similar reduced sensitivity to cisplatin, and co-depletion of SENP1 and JAK2 did not further increase the sensitivity of IGROV1 CR cells to cisplatin compared to depletion of SENP1 or JAK2 alone, indicating that SENP1 and JAK2 function in the same pathway. Ectopic expression of JAK2 in IGROV1 CR cells with depleted SENP1 restored cisplatin resistance of IGROV1 CR cells to the similar levels as cells treated with control siGL2, suggesting that JAK2 is the primary target of SENP1 to promote platinum-resistance in ovarian cancer cells.

Momordin Ic (Mc) is a natural pentacyclic triterpenoid compound that inhibits SENP1 activity with IC_50_ at 15.37 µM in vitro^35^. Inhibition of SENP1 by Mc re-sensitized SKOV3 CR, PEO4 and PEO23 cells to cisplatin (Fig. 7D–F). Significantly, Mc exhibited great synergy with cisplatin to inhibit cell proliferation of resistant cells, as indicated by combination index (CI) (synergism: CI < 1; additive effect: CI = 1; and antagonism: CI > 1). Using clonogenic survival assay, we also found that Mc showed similar synergy with cisplatin in IGROV1 CR (Fig. 7G and H). Together our results suggest that inhibition of SENP1 is a promising therapeutic approach to overcome platinum-resistance in ovarian cancer.

## Discussion

In this study, we have demonstrated that SENP1 controls JAK2 function by regulating its cellular localization via deSUMOylation, and deSUMOylation of JAK2 by SENP1 indirectly regulates JAK2/STAT signaling pathway by shuttling JAK2 between cytoplasm and nucleus. Importantly, we provided evidence to demonstrate that elevated expression level of SENP1 promotes cytoplasmic accumulation of JAK2, resulting in the activation of JAK2/STAT pathway. We have found that activated JAK2/STAT/anti-apoptosis signaling leads to platinum-resistance in ovarian cancer cells^4^. This novel SENP-1-mediated mechanism encouraged us to found that SENP1 knockdown or inhibition overcomes cisplatin-resistance in ovarian cancer (Fig. 7I).

It has been revealed that JAK2 is modified by SUMO2/3 chains to a high molecular mass and this modification induces JAK2 cellular translocation in nucleus^40^. Thus, the SUMO-mediated pathway is essential to regulate JAK2 cellular function. Our study identified the protease that controls this important process. SENP1-mediated regulation of JAK2 may explain why growth hormone-induced nuclear translocation did not increase the size of the nuclear pool of JAK2^45^. JAK2 is a bulky protein with a 131 kDa molecular weight. The size of JAK2 prevents its freely diffuse between cytoplasm and nucleus. Since JAK2 does not have classic nuclear localization sequence^17^, it is still not clear how SUMOylation of JAK2 affects its translocation through the nuclear membrane.

Although SENP1 is overexpressed in many cancer types and is critical for cellular pathways^46,47^, how SENP1 is regulated in cells is poorly understood. Our study for the first time revealed that a transcription factor RUNX2 is responsible for the increased SENP1 mRNA level in cisplatin-resistant ovarian cancer cells. We still don’t know why and how RUNX2 levels are up-regulated in platinum-resistant ovarian cancer cells. Given that ROS levels are increased in platinum-resistance ovarian cancer cells^48^, it is possible that elevated ROS levels may trigger the increased expression of RUNX2.

Anti-SENP1 was regarded as a useful approach for cancer therapy^35,49,50^. However, so far, the development of SENP1 inhibitors has achieved the limited progress. To date, several SENP1 inhibitors have been identified, including benzodiazepine-based peptidomimetic covalent compounds, SUMO-derived peptide-based covalent inhibitors, non-covalent 2-(4-chlorophenyl)-2-oxoethyl 4-benzamidobenzoates and 1-[4-(N-benzylamino)phenyl]-3-phenylureas and in silico screened compound 13m^31,33,34,51,52^. The IC50s of these compounds range from 3.5 to 29.6 µM. Thus, significant improvement of efficacy is required for further clinical application. Among these compounds, Mc has been identified to directly bind and inhibit SENP1 in prostate cancer^35^. This study provides with a potential application of SENP1 inhibitors for the treatment of platinum-resistant ovarian cancer patients.

### Ethics approval

All animal experiments were conducted in accordance with the Institution Animal Care and Use Committee of the George Washington University for laboratory animal use and care, and all relevant ethical regulations were followed. All human samples and studies were approved by the local ethics committees (institutional review board reference No: UW 05-143 T/806 and UW 11-298) and the studies abide by the Declaration of Helsinki principles. Informed consent was received before inclusion in the study.

## Data Availability

RNA-Seq data is available at Gene Expression Omnibus (GSE115939). Mass spectrometry data is available at Figshare (DOI: 10.6084/m9.figshare.12885524). All remaining data are contained within the article.

## References

[1] Kurdi M, Booz GW (2009). JAK redux: a second look at the regulation and role of JAKs in the heart. Am. J. Physiol. Heart Circulatory Physiol..

[2] Quintas-Cardama A, Kantarjian H, Cortes J, Verstovsek S (2011). Janus kinase inhibitors for the treatment of myeloproliferative neoplasias and beyond. Nat. Rev. Drug Discov..

[3] Liu CS, Yang-Yen HF, Suen CS, Hwang MJ, Yen JJ (2017). Cbl-mediated K63-linked ubiquitination of JAK2 enhances JAK2 phosphorylation and signal transduction. Sci. Rep..

[4] Zhou W (2018). Autocrine activation of JAK2 by IL-11 promotes platinum drug resistance. Oncogene.

[5] Gabler K, Behrmann I, Haan C (2013). JAK2 mutants (e.g., JAK2V617F) and their importance as drug targets in myeloproliferative neoplasms. Jak.-Stat..

[6] Ding L (2010). MiR-375 frequently downregulated in gastric cancer inhibits cell proliferation by targeting JAK2. Cell Res..

[7] Hedvat M (2009). The JAK2 inhibitor AZD1480 potently blocks Stat3 signaling and oncogenesis in solid tumors. Cancer Cell.

[8] Britschgi A (2012). JAK2/STAT5 inhibition circumvents resistance to PI3K/mTOR blockade: a rationale for cotargeting these pathways in metastatic breast cancer. Cancer Cell.

[9] Zhao M (2011). JAK2/STAT3 signaling pathway activation mediates tumor angiogenesis by upregulation of VEGF and bFGF in non-small-cell lung cancer. Lung Cancer.

[10] Judd LM (2014). Inhibition of the JAK2/STAT3 pathway reduces gastric cancer growth in vitro and in vivo. PloS One.

[11] Baxter EJ (2005). Acquired mutation of the tyrosine kinase JAK2 in human myeloproliferative disorders. Lancet.

[12] Levine RL (2005). Activating mutation in the tyrosine kinase JAK2 in polycythemia vera, essential thrombocythemia, and myeloid metaplasia with myelofibrosis. Cancer Cell.

[13] James C (2005). A unique clonal JAK2 mutation leading to constitutive signalling causes polycythaemia vera. Nature.

[14] Quintas-Cardama A, Verstovsek S (2013). Molecular pathways: Jak/STAT pathway: mutations, inhibitors, and resistance. Clin. Cancer Res..

[15] O’Shea JJ, Holland SM, Staudt LM (2013). JAKs and STATs in immunity, immunodeficiency, and cancer. N. Engl. J. Med..

[16] Levy DE, Darnell JE (2002). Stats: transcriptional control and biological impact. Nat. Rev. Mol. cell Biol..

[17] Zouein FA, Duhe RJ, Booz GW (2011). JAKs go nuclear: emerging role of nuclear JAK1 and JAK2 in gene expression and cell growth. Growth Factors.

[18] Qian CJ, Yao J, Si JM (2011). Nuclear JAK2: form and function in cancer. Anat. Rec..

[19] Flotho A, Melchior F (2013). Sumoylation: a regulatory protein modification in health and disease. Annu Rev. Biochem.

[20] Matunis MJ, Coutavas E, Blobel G (1996). A novel ubiquitin-like modification modulates the partitioning of the Ran-GTPase-activating protein RanGAP1 between the cytosol and the nuclear pore complex. J. Cell Biol..

[21] Gareau JR, Lima CD (2010). The SUMO pathway: emerging mechanisms that shape specificity, conjugation and recognition. Nat. Rev. Mol. Cell Biol..

[22] Hendriks IA, Vertegaal AC (2016). A comprehensive compilation of SUMO proteomics. Nat. Rev. Mol. cell Biol..

[23] Hickey CM, Wilson NR, Hochstrasser M (2012). Function and regulation of SUMO proteases. Nat. Rev. Mol. cell Biol..

[24] Nayak A, Muller S (2014). SUMO-specific proteases/isopeptidases: SENPs and beyond. Genome Biol..

[25] Yeh ET (2009). SUMOylation and De-SUMOylation: wrestling with life’s processes. J. Biol. Chem..

[26] Cheng J, Wang D, Wang Z, Yeh ET (2004). SENP1 enhances androgen receptor-dependent transcription through desumoylation of histone deacetylase 1. Mol. Cell. Biol..

[27] Cheng J, Kang X, Zhang S, Yeh ET (2007). SUMO-specific protease 1 is essential for stabilization of HIF1alpha during hypoxia. Cell.

[28] Kaikkonen S (2009). SUMO-specific protease 1 (SENP1) reverses the hormone-augmented SUMOylation of androgen receptor and modulates gene responses in prostate cancer cells. Mol. Endocrinol..

[29] Gyorffy B, Lanczky A, Szallasi Z (2012). Implementing an online tool for genome-wide validation of survival-associated biomarkers in ovarian-cancer using microarray data from 1287 patients. Endocr.-Relat. Cancer.

[30] Huang W (2012). Triptolide inhibits the proliferation of prostate cancer cells and down-regulates SUMO-specific protease 1 expression. PloS One.

[31] Chen Y (2012). 2-(4-Chlorophenyl)-2-oxoethyl 4-benzamidobenzoate derivatives, a novel class of SENP1 inhibitors: Virtual screening, synthesis and biological evaluation. Bioorg. Med. Chem. Lett..

[32] Xie W (2016). Development and evaluation of a highly reliable assay for SUMO-specific protease inhibitors. Bioorg. Med. Chem. Lett..

[33] Uno M, Koma Y, Ban HS, Nakamura H (2012). Discovery of 1-[4-(N-benzylamino)phenyl]-3-phenylurea derivatives as non-peptidic selective SUMO-sentrin specific protease (SENP)1 inhibitors. Bioorganic Med. Chem. Lett..

[34] Zhao Y, Wang Z, Zhang J, Zhou H (2016). Identification of SENP1 inhibitors through in silico screening and rational drug design. Eur. J. Med. Chem..

[35] Wu J (2016). Momordin Ic, a new natural SENP1 inhibitor, inhibits prostate cancer cell proliferation. Oncotarget.

[36] Li J, Summerlin M, Nitiss KC, Nitiss JL, Hanakahi LA (2017). TDP1 is required for efficient non-homologous end joining in human cells. DNA Repair.

[37] Heo J (2015). TDP1 promotes assembly of non-homologous end joining protein complexes on DNA. DNA repair.

[38] Vichai V, Kirtikara K (2006). Sulforhodamine B colorimetric assay for cytotoxicity screening. Nat. Protoc..

[39] Chou TC, Talalay P (1984). Quantitative analysis of dose-effect relationships: the combined effects of multiple drugs or enzyme inhibitors. Adv. Enzym. Regul..

[40] Sedek M, Strous GJ (2013). SUMOylation is a regulator of the translocation of Jak2 between nucleus and cytosol. Biochem J..

[41] Ihle JN (1996). STATs: signal transducers and activators of transcription. Cell.

[42] Yuan ZL, Guan YJ, Chatterjee D, Chin YE (2005). Stat3 dimerization regulated by reversible acetylation of a single lysine residue. Science.

[43] Maruyama T, Abe Y, Niikura T (2018). SENP1 and SENP2 regulate SUMOylation of amyloid precursor protein. Heliyon.

[44] Bawa-Khalfe T, Cheng J, Wang Z, Yeh ET (2007). Induction of the SUMO-specific protease 1 transcription by the androgen receptor in prostate cancer cells. J. Biol. Chem..

[45] Lobie PE (1996). Constitutive nuclear localization of Janus kinases 1 and 2. Endocrinology.

[46] Xu Y (2010). Induction of SENP1 in endothelial cells contributes to hypoxia-driven VEGF expression and angiogenesis. J. Biol. Chem..

[47] Sun XX (2018). SUMO protease SENP1 deSUMOylates and stabilizes c-Myc. Proc Natl Acad. Sci. USA.

[48] Meng YX (2018). DUOXA1-mediated ROS production promotes cisplatin resistance by activating ATR-Chk1 pathway in ovarian cancer. Cancer Lett..

[49] Jiang Z (2012). SENP1 deficiency promotes ER stress-induced apoptosis by increasing XBP1 SUMOylation. Cell Cycle.

[50] Cui CP (2017). SENP1 promotes hypoxia-induced cancer stemness by HIF-1 alpha deSUMOylation and SENP1/HIF-1 alpha positive feedback loop. Gut.

[51] Qiao Z (2011). Design, synthesis, and biological evaluation of benzodiazepine-based SUMO-specific protease 1 inhibitors. Bioorg. Med. Chem. Lett..

[52] Albrow VE (2011). Development of small molecule inhibitors and probes of human SUMO deconjugating proteases. Chem. Biol..

